# Strategies for Surface Design in Surface Plasmon Resonance (SPR) Sensing

**DOI:** 10.3390/bios13040465

**Published:** 2023-04-07

**Authors:** Cristina-Virginia Topor, Mihaela Puiu, Camelia Bala

**Affiliations:** 1 Department of Analytical and Physical Chemistry, University of Bucharest, 4-12 Regina Elisabeta Blvd., 030018 Bucharest, Romania; 2 R&D Center LaborQ, University of Bucharest, 4-12 Regina Elisabeta Blvd., 030018 Bucharest, Romania

**Keywords:** biosensor, surface plasmon resonance, immobilization, click chemistry, nanomaterials, metal organic framework

## Abstract

Surface plasmon resonance (SPR) comprises several surface-sensitive techniques that enable the trace and ultra-trace detection of various analytes through affinity pairing. Although enabling label-free, sensitive detection and real-time monitoring, several issues remain to be addressed, such as poor stability, non-specific adsorption and the loss of operational activity of biomolecules. In this review, the progress over sensor modification, immobilization techniques and novel 2D nanomaterials, gold nanostructures and magnetic nanoparticles for signal amplification is discussed. The advantages and disadvantages of each design strategy will be provided together with some of the recent achievements.

## 1. Introduction

Nowadays, rapid detection and diagnosis methods for medical, food safety and environmental monitoring require leading-edge technologies that enable economically viable user-friendly detection systems with fast time response [1,2]. Surface plasmon resonance (SPR) biosensing attracted considerable interest in recent decades and is a technique that allows label-free detection of various analytes and real-time monitoring of biomolecular events. Ongoing efforts have been made to develop biosensors for disease biomarkers such as nucleic acids (DNA, RNA, microRNA), proteins, antibodies, bacteria, cells, and others [3,4,5]. The conventional SPR method requires one binding component to be immobilized on a sensor chip while the other component in the solution is run over the sensor surface [6]. Therefore, one of the imperative parts of SPR-based sensing devices is sensor chip modification because it can greatly influence sensing performance. Versatile chemical activation of the surface and ligand immobilization has been often reported in the literature. Usually, the first step to design a biosensor is to bind a linker to the gold surface to obtain a functionalized surface, such as alkanethiols, dextran polymers or silane-modified layers; next, a certain ligand is immobilized to the modified surface, that specifically interacts with the target analyte in solution. Additionally, several strategies have been proposed to enhance the SPR signal to increase the sensitivity and specificity towards the target analyte.

Several challenges still affect SPR-based detection, such as low sensitivity in complex matrices, non-specific interactions, protein fouling or steric hindrance effects occurring at the active binding sites. To overcome these limitations, metallic or magnetic nanoparticles and 2D nanomaterials with outstanding optical and conductive properties have been reported to achieve excellent results [7].

**Scheme 1:**
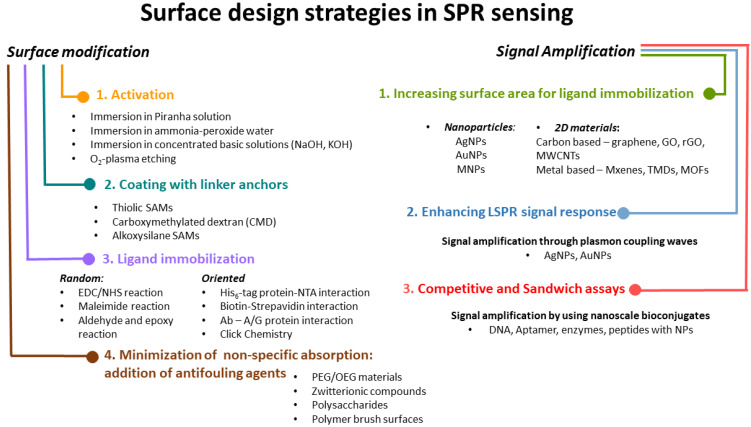
Selection of design strategies for surface modification of the SPR chip discussed in this review.

In this paper, we will briefly review the main strategies for designing binding formats and ligand anchoring, focusing on the most recent trends for surface modification and signal enhancement in SPR binding assays. The conventional methods of covalent immobilization that are successfully used today will be briefly described, while the emphasis will be placed on the use of nanocomposite materials for coating gold surfaces that exceptionally enhance the performance of the SPR sensor in terms of sensitivity, stability, and reusability. The most relevant topics of this review are summarized in Scheme 1.

## 2. SPR Operating Principle

**Figure 1:**
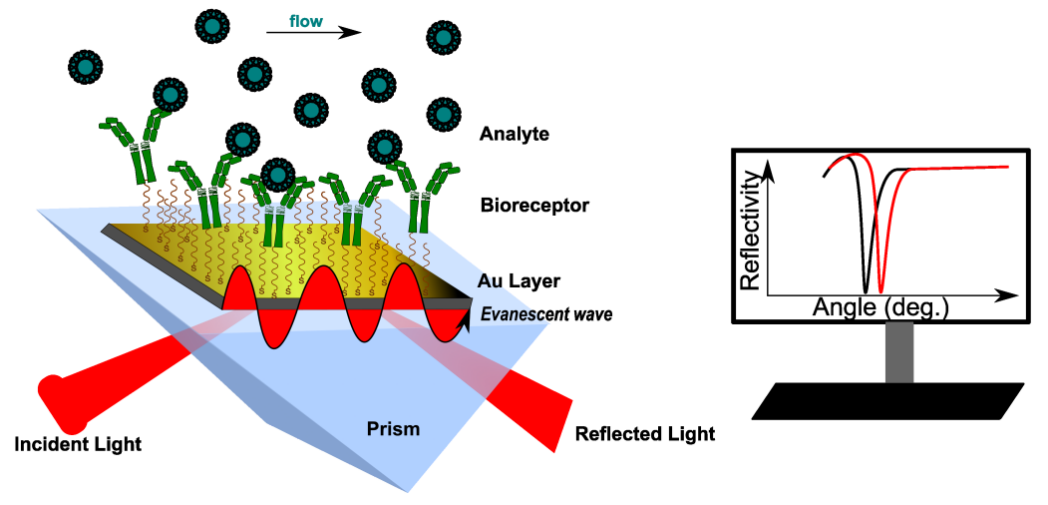
Principle of conventional SPR biosensor with Kretschmann configuration. The analyte binding to the immobilized bioreceptor causes a change in RI near the surface, recorded as the shift of the resonance angle (here, measured at a fixed wavelength). RI reaches a constant value at equilibrium.

Surface plasmon resonance (SPR) is an optical method that measures changes in the refractive index (RI) of the medium in close proximity to a metallic surface, caused by binding events occurring at the surface [6]. Most SPR devices use the Kretschmann configuration, where an incident polarized light beam goes through a prism on a sensor chip, which consists of two parts: glass and a metal layer (usually gold) [8,9]. Surface plasmons (SPs) are generated because of the coupling of the incident oscillating electric field of the electromagnetic (EM) wave with the electrons at the metal/dielectric interface. When an SP couples with a photon, the resulting excitation is called surface plasmon polariton (SPP) [10]. The incident light, after total internal reflection (TIR) within a glass prism, generates an electric field on the interface’s opposite site. The SPPs also create an electric field that extends into the medium on either side of the metallic film. This field is called an evanescent wave (EW) because the amplitude of the wave drops exponentially with the distance from the interface. The evanescent field wave and the incident light have the same wavelength. At a specific angle (SPR angle, φ), the SPPs can be excited to resonate for a given monochromatic light. In specific conditions, φ depends only on the refractive index (RI) of the medium (or the dielectric constant) [11]. This causes a sharp dip in the intensity of the reflected light (Figure 1). The most used sensing configurations are prism, grating, waveguide and fiber-optic, with two ways to excite the surface plasmons: attenuated total reflection (ATR) and diffraction.

The sensitivity of an SPR sensor is defined as the ratio between the change in sensor output and the change in the refractive index. Thus, the response of the SPR sensor medium is sensitive to changes in the refractive index of the medium near the surface, changes that may be caused by affinity pairing, chemical reaction, adsorption/desorption, temperature, and pH [12]. The SPR signal can be measured according to three main methodologies: angular, wavelength, and intensity (amplitude) [10]. In a typical angular scanning SPR experiment, monochromatic light is directed to the metal surface at several incidence angles. The excitation of SPs causes light absorption, recorded as a dip in the angular spectrum of the reflected light [13]. Data are further processed either by angular scanning of the incident light or by sensorgrams (angle shift versus time). In an intensity-modulated SPR experiment, RI variations are monitored as changes in the resonance intensity, at a fixed incidence angle and wavelength. This methodology is suitable for obtaining images with SPR sensors, and it is commonly called SPR imaging (SPRi) [14]. In wavelength-modulated SPR measurements, the angle of the incident light is settled at a certain value, whereas the wavelength of the reflected light is adjusted [9]. The reflected intensity dip is measured against the change in the refractive index over a range of incident wavelengths, and the wavelength with the strongest coupling is used as a sensor output.

Multi-parametric SPR (MP-SPR) was reported recently to enhance the SPR signal. In this case, two or more wavelengths were used; the thickness and refractive of different modified layers were determined by modelling the SPR signal at each wavelength [15,16].

In a common SPR experiment, the SPR cell works either in batch or flow configuration; either way, the angle shift due to the surface modification is then plotted versus time. Batch configuration is less used because it requires longer time frames for signal stabilization. However, miniaturized instruments such as fiber optic surface plasmon resonance sensors (FO-SPR, where the prism is replaced by the core of an optical fiber) are better suited for monitoring interactions between immobilized ligands and different bacterial strains, similar to in vivo conditions [17]. On the other hand, flow conditions are mostly used for real-time monitoring and the determination of kinetic parameters.

## 3. Surface Modification in SPR Assays: Coating Strategies

In SPR assays, the key stage is to modify the metal surface with different coatings, preparing the SPR chip for ligand immobilization. Before any use, the metallic surface should be cleaned and activated. The common substrate for SPR sensors is gold, due to its chemical stability and resistance to corrosion. Besides that, compounds containing thiol groups can be spontaneously deposited onto the surface, via gold–thiol chemistry. Some studies reported the use of silver as an SPR metal layer, but the main drawbacks were the chemical instability and fast oxidation following air exposure [18]. For these reasons, gold is the preferred substrate in SPR assays.

### 3.1. Surface Activation

Generally, before coating with an organic layer, the metal must be chemically activated by removing all the contaminants (inorganic or organic) and allowing further modification. The most common surface pre-treatments are (a) immersion in piranha mixture (H_2_SO_4_/H_2_O_2_); (b) immersion in concentrated NaOH solution (2.5 M); (c) immersion ammonia-peroxide water mixture (NH_4_OH/H_2_O_2_/H_2_O); and (d) O_2_-plasma etching [19]. The roughest surfaces were obtained after piranha and ammonia-peroxide water treatments. Moreover, it was observed that the piranha solution provided the highest adsorption degree of organophosphorus acids. The disadvantages of using piranha solution for gold chip activation are changes in the morphological structure of the gold, an increase in surface hydrophilicity through the addition of hydroxyl groups, and finally, after more than one exposure, the occurrence of defects and breakdown of the gold film. On the other hand, oxygen plasma has been shown to remove organic contaminants just as well as the piranha mixture, and it can be used more than once [20]. It also led to a smoother surface, with a uniform structure. After activation, linker molecules are deposited onto gold. Several common linkers serve different purposes, as will be shown further.

### 3.2. Thiol Anchoring

**Figure 2:**
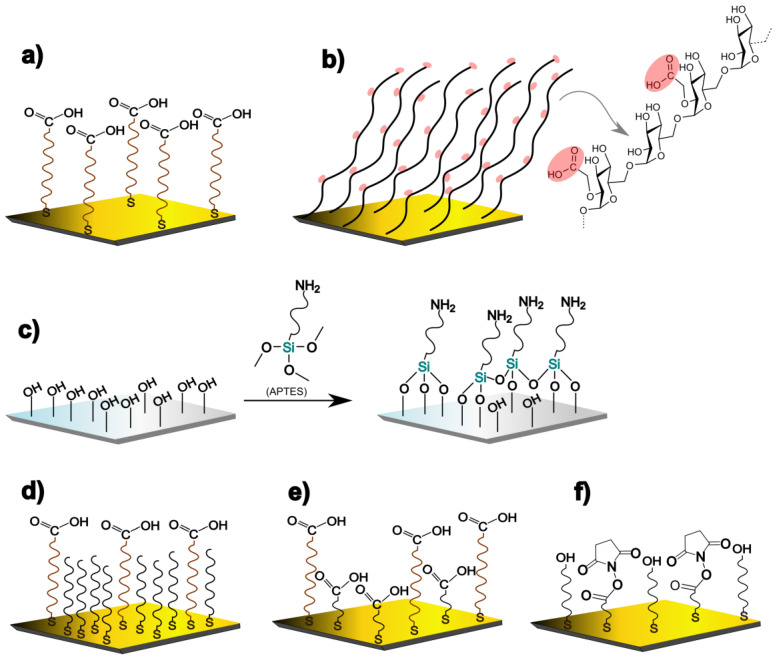
Schematic representation of chemically modified gold surfaces for SPR experiments: (**a**) alkanethiol self-assembled monolayer (11-mercaptoundecanoic acid); (**b**) dextran-modified polymeric layer (carboxymethylated dextran); (**c**) silicon-modified glass-based chip (silanization reaction via APTES); modified thiol surfaces with mixed species for avoiding non-specific interaction; (**d**) 11-MUA/1-octanethiol (adapted from [22] with permission of Elsevier); (**e**) 11-MUA/3-mercaptopropionic acid (adapted from [24]). This is an unofficial adaptation of an article that appeared in an ACS publication. ACS has not endorsed the content of this adaptation or the context of its use; (**f**) DSP/MCH. Adapted from [25] with permission of Wiley.

It is well known that gold possesses a high affinity for thiols, and the deposition of thiols on the surface is followed by the formation of self-assembled monolayers (SAMs). SAMs are usually the result of the spontaneous alignment of alkanethiols on gold. Different types of alkanethiol can be used, depending on the nature of terminal groups (-CH_3_; -COOH; -NH_2_; -OH), the number of methyl groups, and the aromatic or aliphatic structure [21]; 11-mercaptoundecanoic acid (11-MUA), represented in Figure 2a, is the most used linker because of its hydrophilic nature. Moreover, the carboxyl end group can easily react with primary amine groups from antibodies [22] and proteins [23] via EDC (1-Ethyl-3-(3-dimethyl aminopropyl)carbodiimide)/NHS (N-hydroxysuccinimide) esters. A viable strategy for obtaining a larger surface contact with bioreceptors is the use of mixtures of 11-MUA and short-chain thiols such as 1-octane thiol [22] or 3-mercaptopropionic acid [24] (Figure 2d,e). Ataman Sadik et al. reported a mixed self-assembled monolayer composed of 3,3′-dithiodipropionic acid di(N-hydroxysuccinimide ester) (DSP) and 6-mercapto-1-hexanol (MCH) that reduced the steric hindrance and minimized the non-specific interactions (Figure 2f) [25]. Even if DSP and MCH have the same length, the binding degree of bovine serum albumin (BSA) and lysozyme to DSP was higher. The same research group has developed an immunosensor for thrombin by immobilizing anti-thrombin antibodies on the DSP/MCH layer. The linear range was 1.0–500.0 nM [25,26]. However, there are some disadvantages of metal coating with thiol-SAMs. First, SAMs are not stable when they are stored for a long period at room temperature. There is also a risk that the thiol groups may be oxidized. Furthermore, thiol anchoring is a time-consuming process, requiring at least 12 h.

Another approach is the direct attachment of a biomolecule of already modified thiol group at one end to the gold chip. Wang et al. [27] studied the direct attachment of a specific aptamer for detecting *Escherichia coli* (*E. coli*) and *Staphylococcus aureus* (*S. aureus*). They studied the thickness of the immobilized short-chain aptamer in the presence of different concentrations of NaCl and observed that at a lower concentration, the aptamer thickness increased. Moreover, the addition of NaBr led to an increase in the aptamer density. Droz et al. studied three different strategies to immobilize ssDNA on gold: thiol (DNA-SH) chemosorption, dithiocarbamate (DNA-DTC) chemisorption, and physical absorption. MCH was added afterwards to reduce the non-specific adsorption and block the pin-hole defects. From all three, DNA-SH showed the highest stability in both homogeneous and MCH mixed layers, while DNA-DTC was prone to desorption in the presence of MCH [28]. Simon et al. deposited a peptide-nucleic acid (PNA) layer by micro-spotting thiolated PNA directly to bare Au. They monitored the high-affinity interaction between PNA and microRNA. It was observed, by comparing the PNA strands prehybridized with a complementary DNA strand and the ssPNA, that the prehybridized PNA strands exhibited a wide dynamic range for microRNA and the lowest amount obtained was 140 fmol [29]. Direct attachment of biomolecules on gold surfaces showed some disadvantages such as non-specific interactions and physical adsorption, but the one-step immobilization is less time-consuming and involves fewer reagents.

### 3.3. Dextran-Based Hydrogel Attachment

Carboxymethylated dextran (CMD) is often used for surface modification, especially for protein immobilization. Compared with SAMs, carboxymethylated dextrans are not strictly oriented, offering a higher degree of freedom for interactions with bioreceptors. Dextran is a branched polysaccharide modified with carboxymethyl groups (represented in Figure 2b) that can easily react with free-amino functional groups of a biomolecule. CMD hydrogels present some interesting properties. Because of their ramified structure, more functional groups are available for interaction. Moreover, they have a 3D structure, which can be correlated to a larger surface and more binding sites. Another advantage associated with their structure is the minimization of non-specific binding [30]. Compared with SAMs, which are thin layers (less than 2 nm), the thickness of CMDs is between 100 and 200 nm, which can lower the SPR response and sensitivity [31]. Another drawback is that the functional groups from the CMD are not distributed uniformly, and this can hinder the linkage with the bioreceptor. The most common CMD-modified chips are available commercially from Biacore (CM5) [32,33].

### 3.4. Silane Attachment

Silicon-based biosensors are based on alkoxysilane SAMs. The silanization reaction takes place only if the surface has been previously treated to contain hydroxyl groups. Silane layers can be added to different surfaces such as glass, silicon, or gold [34,35,36]. SPR substrates covered with silicon oxides (SiO_2_) or silicon nitrides are preferred because of the ease and cost-effectiveness of their processing. First, the surface needs to be activated to obtain hydroxyl groups for further reactions with silanes. Afterwards, the silane is added to bind (3-aminopropyl)triethoxysilane (APTES), which is a silane coupling agent [37,38]. APTES has one end-functionalized with an amino group, while the other end is a silane group (Figure 2c). The thickness of the silane layer grows with the concentration of added APTES [36]. Moreover, different end groups can be attached to the silane such as aldehydes, cyano (C=N) groups, thiols, poly(ethylenglicol) (PEG), carboxylic acids and others [39].

## 4. Ligand Immobilization onto Coated SPR Chip

One of the most important parts of designing a biosensor is the attachment of a ligand a molecule that has an affinity or a specific interaction with the target analyte to the modified SPR chip. For this purpose, a broad range of biomolecules are used, such as aptamers, antibodies, nucleic acids, enzymes, or peptides [40,41,42,43,44], all of them having different functional groups (often free-amino groups) able to react with other functional groups from the modified surface. Of note, there are other parameters such as the pH, temperature and concentration that can affect the interaction and should be considered.

### 4.1. Random Covalent Immobilization via EDC/NHS Esters

**Figure 3:**
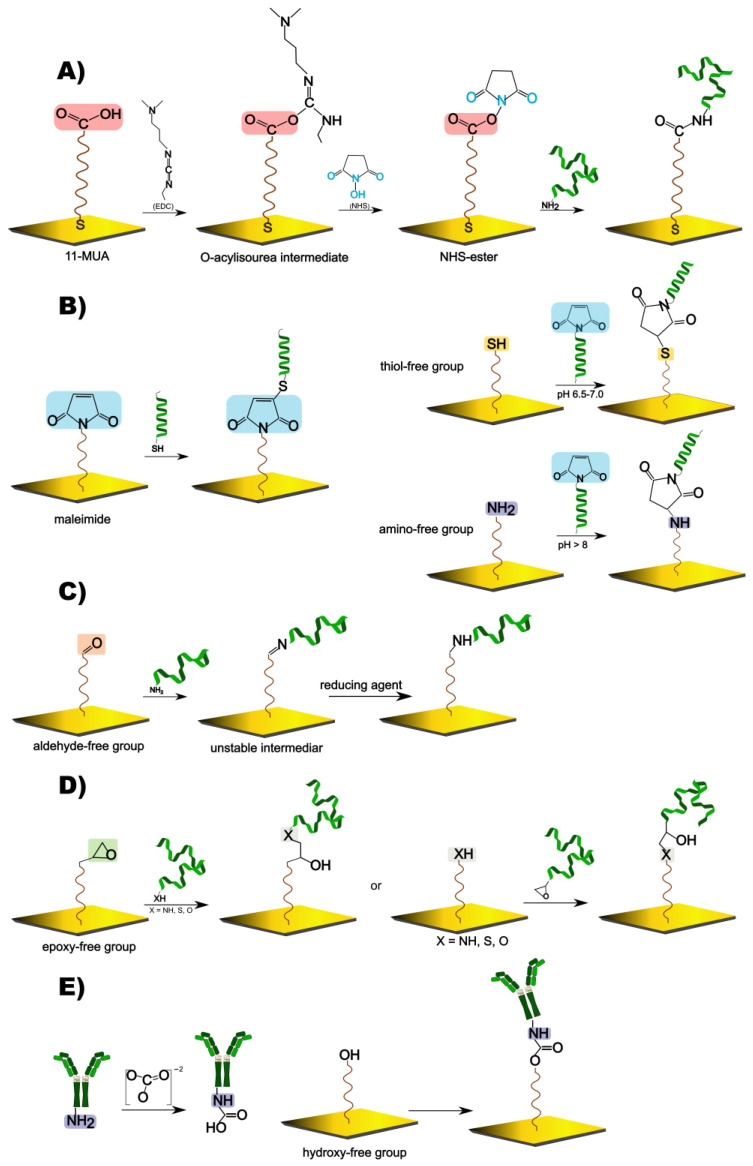
Random covalent immobilization strategies: (**A**) EDC/NHS immobilization. The EDC/NHS chemistry has 2 main steps. First, the carboxyl group is activated by EDC and is turned into an O–acylisourea, an unstable intermediate. NHS reacts fast with this intermediate, forming an active NHS-ester that subsequently reacts with the primary amine groups of the ligand; (**B**) maleimide reaction with thiol or primary amine groups; (**C**) aldehyde reaction with available amine group; (**D**) epoxy reaction with an available amine, thiol, or hydroxyl group; (**E**) amine reaction with an available hydroxyl group in presence of carbonate.

Once the surface has been modified with functional groups, several strategies for ligand immobilization have been considered. EDC/NHS coupling reaction is the most common: the carboxyl (COOH) group is converted in an active NHS ester able to form an amide bond with the primary amine from the ligand (Figure 3A) [45,46]. This strategy is usually used for MUA or dextran-covered surfaces because they already contain carboxyl groups.

### 4.2. Random Covalent Binding through Maleimide Reaction

Another interesting approach is the reaction of a surface-bound maleimide with an exposed thiol group of a protein or a modified nucleic acid (Figure 3B). The maleimide has a similar structure to the NHS-ester, but in this case, an unsaturated imide binds the free SH group. Moreover, this strategy can be used in reverse, by having a thiol functional group available on the surface and by reacting with a modified maleimide from a linker or a biomolecule [47,48]. Additionally, the maleimide can react with an amino group as well, upon a similar reaction mechanism as with the thiol group. In this case, it is very important to control the pH, because at a higher pH (>8), the maleimide will be more reactive towards primary amines [49].

### 4.3. Random Covalent Reactions Involving Aldehyde and Epoxy Groups

Aldehyde-modified SPR surfaces can react with an N-terminal amino acid from a protein, forming an unstable imine intermediate that is further reduced to a stable amine. Other reactive groups of interest are epoxy and hydroxy (Figure 3C). These groups are often found on oxidized graphene sheets (GO), which are used to increase the surface density of the bound ligand [50]. Epoxides react with the available amine, thiol or hydroxyl functionalities from biomolecules; hydroxyl groups form carbamates in the presence of carbonate and the amino group from the ligand (Figure 3D,E) [37,51,52,53].

### 4.4. Oriented Immobilization Strategies

**Figure 4:**
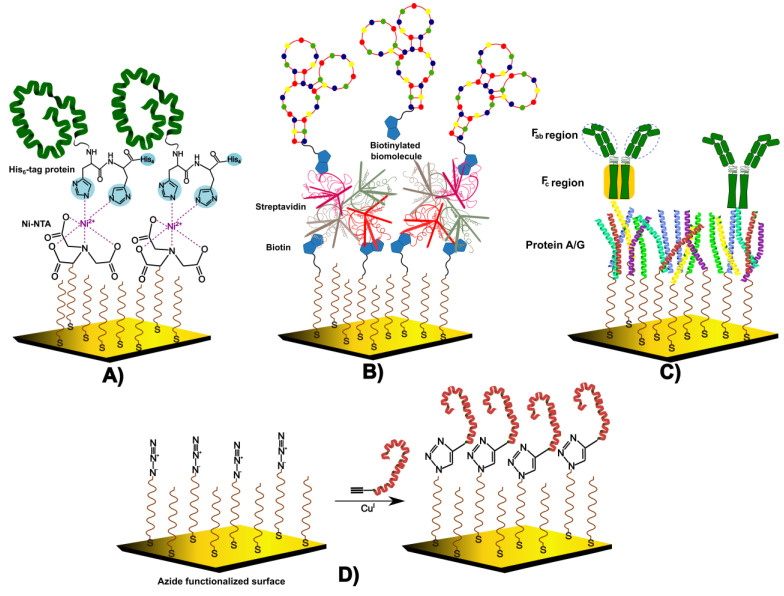
Strategies for oriented immobilization: (**A**) surface-immobilized NTA reacts with Ni^2+^. In the second step, the chelate complex binds the His_6_-tag protein; (**B**) biotin–streptavidin interaction between the immobilized streptavidin and biotinylated aptamer; (**C**) antibody immobilization on the SPR chip via protein A/G, which specifically binds to the F_c_ antibody region; (**D**) click chemistry: the surface is modified with azide groups that subsequently react with the alkyne modified ligand.

Oriented immobilization of biomolecules on SPR chips meets several challenges, especially when no information is given about the arrangement and distribution of the ligands (proteins, antibodies, and aptamers) on the surface. To achieve an oriented binding of proteins, nitrilotriacetic acid (NTA) is used in the so-called His_6_-tag protein immobilization (Figure 4A). NTA forms a chelate complex with the transition metal ion (Ni^2+^, Co^2+^, Cu^2+^, Zn^2+^); afterwards, His_6_-tag protein (which has six histidine tags) binds to the ion with two imidazole groups of histidine [54] biomolecules in the presence of Cu(I) catalyst). Biotin–streptavidin noncovalent immobilization is often used in the oriented binding of antibodies. Usually, streptavidin (which consists of four monomer units) is added to an MUA or a dextran matrix, with free carboxyl groups that are subsequently activated via EDC/NHS reaction. The next step is to bind the modified biotinylated biomolecule with the streptavidin (Figure 4B). Even if this method is currently used for antibodies, several recent studies have reported the immobilization of glycans or aptamers. For example, a glycan–protein interaction was investigated by immobilizing glycans on the streptavidin-biotin substrate [55]. A similar approach was used to obtain an aptasensor for *Brucella melitensis* bacteria. In this case, a modified biotin–gold chip was covered with avidin and a biotinylated specific aptamer [3]. The main disadvantage of this coupling is that non-specific binding can occur.

Another technique to immobilize antibodies is to exploit antibody–protein A/G interaction. This binding is an oriented one because protein A and protein G bind to IgG, especially to the Fc sequence (the fragment crystallizable region), which is the tail of the antibody (Figure 4C). Because of this, the Fab (the fragment antigen-binding region) is set to interact with the antigen [56]. Parkkila et al. [57] proposed a protein A/G functionalized SPR biosensor for capturing extracellular vesicles (EVs). The A/G protein was immobilized on a modified NHS substrate, while the bound antibody was conjugated to streptavidin. Finally, biotinylated lipid-based nanoparticles were added and the SPR response was investigated. Sometimes, only the G protein is used as an oriented affinity molecule. For example, the detection of two biotoxins (ricin and abrin) was carried out in a sandwich format between the G-protein-bound monoclonal antibody and the biotoxin-specific antibody; then, the analyte of interest was detected [58]. Additionally, to improve the performance of the biological sensors, A and G proteins were bound to different coating layers such as carbon disulphide (CS_2_) or graphene oxide sheets (GO) [59,60].

Click chemistry is another cross-linking reaction that occurs between an alkyne and a functionalized azide in the presence of Cu (I) (Figure 4D). The main advantages of this reaction are operational stability and a good yield. However, there are some limitations, such as that the modification of the ligand with alkyne or azide groups is time consuming [61,62].

### 4.5. Minimizing Non-Specific Adsorption: Addition of Anti-Fouling Agents

Non-specific adsorption is another problem that should be avoided in SPR experiments. Several compounds were reported to show good anti-fouling properties due to the presence of hydrophilic functional groups such as PEG, zwitterionic compounds, polysaccharides, polymer brush surfaces, polyelectrolyte multilayers and others [63]. Poly(ethylene glycol)/oligo(ethylene glycol)—(PEG/OEG) materials are extensively used, because of their capacity of providing resistance to protein adsorption. PEG materials can be functionalized with different reactive groups able to react with the ligand. However, some parameters must be optimized to achieve satisfying surface densities of the grafted PEG such as molecular weight, concentration and chain length (nm) [64]. A comparative study of PEG attached to a gold chip and two other linkers (lipoic acid and 3,3′-dithiobis(sulfosuccinimidyl propionate) was carried out using BSA as a probe for protein fouling. The obtained results showed significant anti-fouling properties of the PEG-modified surface, even at high concentrations of BSA (up to 50 mg/mL) [65].

Zwitterionic compounds belong to an alternative class of polymers that contain equal amounts of cationic and anionic groups. Therefore, the zwitterionic compounds show strong hydration due to the highly charged groups. They can form self-assembled monolayers, with ordered structures that can be tuned [66]. It was reported that polymeric zwitterionic brushes (with thicknesses ranging from 20 to 40 nm) reduce the adhesion of proteins and cell attachment [67].

## 5. Strategies for Signal Amplification

### 5.1. Gold and Silver Nanostructures for Surface Modification in Localised Surface Plasmon Resonance (LSPR) Experiments

The performance of SPR optical biosensors can be increased by adding different nanostructures for signal amplification. Thus, localized surface plasmon resonance (LSPR) occurs on metallic nanostructures (usually gold nanomaterials) that give oscillations of the electrons at their surface. In the case of SPR-based transducers, the charge density oscillations propagate along the metal/dielectric interface, while in localized SPRs (LSPRs)-based transducers, the charge density oscillations are confided on nanoscale metallic structures (disks, rods, spheres, etc.). These metallic nanomaterials have many conducting free electrons that are displaced from their nuclei when an electromagnetic field is applied. At the same time, the opposite charges bring back the electrons at the initial position. Because of this behaviour, the collective oscillations of free electrons increase at a specific frequency. It should be highlighted that plasmons can be excited by incident electromagnetic waves only when the frequency of waves and plasmons coincide [68,69]. Moreover, the sensitivity of LSPR can be influenced by the size of nanoparticles. An LSPR study made with gold nanospheres of different sizes (from 10 nm to 50 nm) showed that the increase in the LSPR peak intensity is correlated with the increase in the particle size. Additionally, their behaviour was studied at different temperatures (from 100 K to 700 K). The best LSPR absorption spectra were obtained at low temperatures [70].

It was also noticed that the shape of nanoparticles plays an important role in LSPR signal enhancement. Different shapes present different electric intensities when the interaction with the incident light occurs. Xu and Geng concluded in their review that nanoparticles with high symmetry such as spherical ones do not provide high sensitivity. On the other hand, when the shape is changed to be less symmetrical (triangle, star, disk, etc.), the electric field intensity is highly improved [71]. Unfortunately, the main limitation is that nanoparticles with less symmetry are more difficult to synthesize and the reproducibility is sometimes difficult to attain.

Plasmonic nanoprobes consisting of gold nanoparticles (AuNPs) and biomolecules are used due to their LSPR properties. In 2018, Kim et al. proposed a sensitive sandwich assay to detect the surface antigen of the hepatitis B virus (HBsAg). They used a glass substrate fabricated with AuNPs, and the antibody was immobilized on the surface of AuNPs. After the capture of HBsAg, a second antibody modified with AuNPs was added in a sandwich assay. The biosensor was able to detect a concentration as low as 100 fg/mL for HBsAg by using an antibody conjugated to 15 nm AuNPs [72].

Gold nanorods (AuNRs) present good optical characteristics due to their particular shape. Plasmon oscillations in AuNRs have two main directions: through their width or length (transversal or longitudinal). An LSPR biosensor based on AuNRs was developed to detect CRP (C-reactive protein). AuNRs were modified with thiol-activated aptamers that interacted further with the target protein. The highest load of aptamer on AuNRs was obtained for a concentration of 20 nM, while the limit of the detection (LOD) for CRP was 25 ng/mL [73].

**Figure 5:**
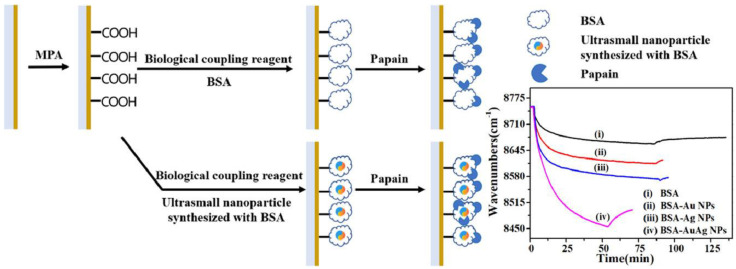
Schematic representation of the modified chip using BSA coated with ultrasmall u/AgNPs $\left(1-3\;\mathrm{nm}\right)$. BSA and BSA-Au/AgNPs bioconjugate were immobilized onto a 3-mercaptopropionic acid (MPA) SAM and allowed to interact further with papain. The binding of papain to the surface-immobilized BSA-Au/AgNPs provided the highest wavenumber shift. Reproduced from [78] with permission of Elsevier.

Gold nanostars (AuNSts) belong to another class of nanoparticles with anisotropic properties caused by their morphology. AuNSts are characterized by large surfaces due to their sharp star structures, and they can be used as materials for surface-enhanced Raman spectroscopy (SERS) [74]. An interesting comparative study was made between the performances of AuNPs (spherical) and AuNSt in the detection of target proteins. Biotinylated AuNPs and AuNSt with similar dimensions were used to interact with avidin, and the subsequent shift changes were measured. It was observed that the highest shift change was caused by AuNSts. Due to their structure and morphology, AuNSt can produce larger plasmonic spectral shifts. AuNSt as clusters presented better optical properties compared to AuNSt alone [75]. Silver nanoparticles (AgNPs) are also used for enhancing the SPR signal. AgNPs have unique optical properties, and their applicability was exploited in many areas such as catalysis, biomedicine and drug delivery [76]. Parit et al. synthesized AgNPs from medicinal plants using nitrate gold as a precursor. The size and shape were characterized. A band at 426 nm in the UV–visible spectrum was obtained, which indicated a successful synthesis of AgNPs. Additionally, as the reaction was complete, the SPR band stabilized at 430 nm. This peak was correlated to the spherical structure of the nanoparticles [77]. Liu et al. developed a biosensor for the detection of the enzyme papain based on ultrasmall nanoparticles of Au and Ag (around 2 nm). Firstly, BSA was employed as a template to synthesize of ultrasmall gold, silver, and bimetallic gold–silver metal nanoparticles (BSA-Au NPs, BSA-Ag NPs, and BSA-Au-Ag NPs, respectively). Then, the BSA-stabilized ultrasmall nanoparticles were immobilized on a gold SPR chip and used further for papain detection. A comparison was made between three types of coating (BSA-AuNPs, BSA-AgNPs and BSA-Au/AgNPs), and it was observed that the SPR sensitivity increased significantly when Au and Ag NPs were used (Figure 5). This behaviour could be explained by the fact that ultrasmall metal nanoparticles could couple with the evanescent waves of the SPR from the chip and enhanced the SPR response [78].

Wang et al. [79] developed a biosensor for microRNA detection. For this, the gold disk was first modified with a DNA-hairpin structure, and MCH has added afterwards. MicroRNA was injected into the flow cell and formed a complex with already immobilized MCH. The next step was the addition of two other DNA probes to form a long and stable dsDNA chain. AgNPs were added to the system to improve the SPR sensitivity. In the presence of AgNPs, dsDNA intercalates with the silver ions, causing a shift in the SPR angle. This protocol presented a linear detection range detection for microRNA of 0.001–0.1 pM and a limit of detection (LOD) of 0.35 fM.

### 5.2. Magnetic Nanoparticles (MNPs) Bioconjugates for Signal Enhancement in SPR Assays

Magnetic nanoparticles (MNPs) exhibit strong magnetic properties. MNPs are composed of a metal and their oxides, most often iron oxides due to their biocompatibility, magnetic features, high surface area and volume ratio. MNPs display good stability and less toxicity than other metallic nanoparticles, which have increased in popularity and in the variety of applications in fields such as catalysis, biosensing, medicine and environment [80]. However, their performance in SPR signal enhancement is explained by the fact that they cause strong changes in refractive index near the Au surface. The binding of MNP bioconjugates to immobilized ligands amplifies the SPR signal in competitive and sandwich assays. Magnetic nanobeads are also used to immobilize biomolecules (often antibodies) onto gold surfaces, and they provide high loads of immobilized ligands, as will be shown further.

An SPR biosensor based on iron oxide nanoparticles was made by Dolci et al. [81]. First, Fe_3_O_4_ nanoparticles were attached covalently on the Au chip, followed by biotin grafting on the surface via copper-catalyzed azide–alkyne cycloaddition (CuAAC) (click chemistry). The efficiency of the prepared nanoparticles was evaluated by monitoring the SPR wavelength shift following the absorption of streptavidin. Their approach consisted of using high-refractive-index dielectric materials such as Fe_3_O_4_ to increase the sensitivity factor of a gold thin film. The resonance peak was shifted to much longer wavelengths than in the absence of nanoparticles. Fe_3_O_4_ nanoparticles modulated the resonance peak of the gold substrate to longer wavelengths at which sensitivity was enhanced. When 15% Fe_3_O_4_ was used, a sensitivity factor of 1885 nm/RIU was obtained (the sensitivity factor was plotted versus incident wavelength). Increasing the amount to 100% Fe_3_O_4_, the sensitivity factor increased almost three times (5200 nm/RIU), showing that MNPs greatly improved the sensibility of the biosensor. In another SPR binding assay, Ochratoxin A was detected through MNPs conjugate antibodies immobilized on an SPR chip using glutaraldehyde as cross-linking agent [82]. Thus, the gold chip was modified using bovine serum albumin conjugate with a glutaraldehyde–thiolamine linker. This created a layer that minimized the non-specific adsorption of OTA. OTA was detected in a linear range from 1–50 ng/mL with a limit of detection of 0.94 ng/mL.

MNPs were also used as a signal amplification material for estradiol (E_2_) detection [83]. An immunosensor using indirect competition was designed, composed of a chitosan and E_2_-BSA layer immobilized on the chip and samples with different concentrations of modified MNPs E_2_-mAb (monoclonal antibody). With the decrease in the E_2_ concentration, the SPR signal increased due to the binding of more E_2_-mAB-MNPs. The biosensor was compared with ELISA, and the results showed a wider linear range for the SPR chip (3.906 to 1000 ng/mL).

Li et al. proposed an immunosensor for detecting the carbendazim (MBC) pesticide using the indirect competition method. For this, they modified a CMD gold layer with MBC–BSA. Then, a mixture of MBC and Au/Fe_3_O_4_ conjugated with MBC antibody was added to the cell flow. To check if Au/Fe_3_O_4_ nanocomposite amplified the SPR signal, a sample without the nanocomposite was made for comparison. When the SPR biosensor was without Au/Fe_3_O_4_, the obtained LOD for MBC was 2.81 ng/mL, while in presence of the gold and metal nanoparticles, the LOD was 0.44 ng/mL [84].

**Figure 6:**
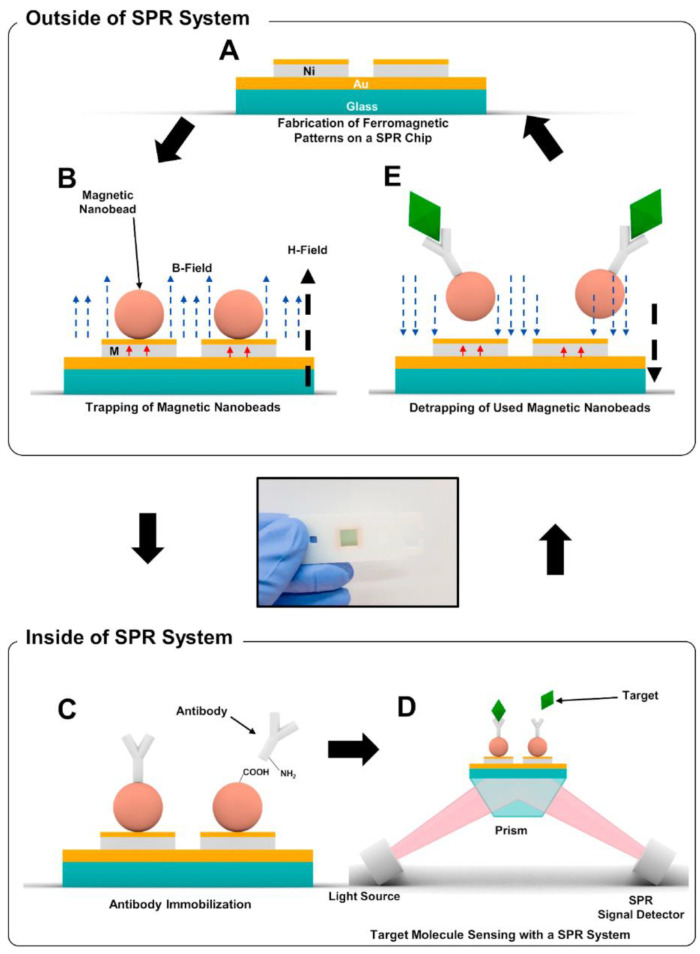
The detection principle of the ferromagnetic patterned SPR sensor chip. (**A**) A reusable SPR chip including ferromagnetic nickel patterns on a conventional SPR chip structure. (**B**) Trapping of magnetic particles on the reusable SPR chip through an external magnetic field. (**C**) Immobilization of antibodies on magnetic particles via EDC-NHS chemistry. (**D**) Detection of target molecules. (**E**) Removal of magnetic particles by applying an external magnetic field in an opposite direction to that for trapping. Reproduced from [85] with permission of Elsevier.

Yoo et al. recently developed a reusable magnetic SPR sensor chip for detecting the H1N1 influenza virus repeatedly in a conventional SPR system [85]. Here, Ni/Au ferromagnetic patterns were fabricated by photolithography and thermal evaporation on a conventional SPR gold chip. After a sensing experiment, the used magnetic particles were removed by external magnetic fields, and a new layer of magnetic particles was immobilized to the SPR sensor chip for additional sensing measurements. Since magnetic particles were trapped on the ferromagnetic patterns the reusable SPR chip was implemented in a traditional SPR system without any applied magnetic fields. The detection principle is described in Figure 6.

### 5.3. 2D Materials for Increasing Surface Loading of Biomolecules

Two-dimensional (2D) nanomaterials are promising elements in optical biosensors. They belong to a new class of nanomaterials with sheet-like structures and transverse dimensions larger than 100 nm, with a typical thickness of less than 5 nm [86]. Their interesting properties include a large surface area and good charge transfer properties, which provide better performance and stability. The evanescent wave crossing through two media with different RIs, the gold chip and the 2D material, couples with the oscillations of the collective electrons, thus enhancing the SPR signal [87,88].

**Figure 7:**
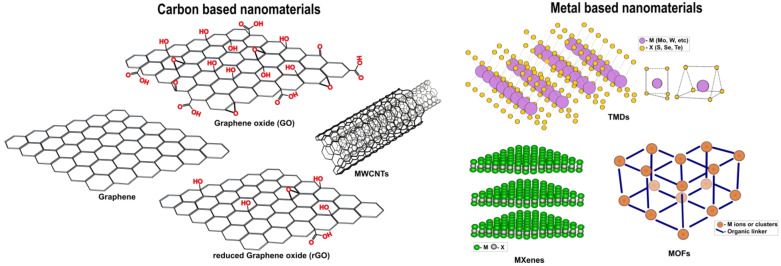
Various 2D (graphene- and metal-based) nanomaterials for sensitivity enhancement in SPR assays.

Two-dimensional materials, as the name suggests, have a 2D layer, and this class of compounds usually consists of two main groups: carbon-based materials such as graphene, graphene oxide (GO), reduced graphene oxide (rGO), and multi-wall carbon nanotubes (MWCNTs) and metal-based nanomaterials such as transitional metal mono/dichalcogenides (TMDs), MXenes, metal–organic frameworks (MOFs), etc. (Figure 7).

Graphene has unique material characteristics. It is a single-crystalline compound that exhibits good electrical and thermal conductivity and optical properties, being formed of sp^2^ carbon hybridized planar sheets (all with the same atom thickness) in a honeycomb shape. Unfortunately, adsorbed impurities or residues on the surface can influence electron transfer, showing a decreased signal for biomolecule sensing [89]. Oxidation of the graphene leads to graphene oxide (GO), which contains oxygen atoms bonded with some carbon atoms (-OH, -COOH, =O, -O-). This functionalized graphene would now have a hydrophilic nature and could be easily dissolved in a protic solvent such as water. By reducing GO with different reducing agents such as alcohol or phenol, reduced graphene oxide (rGO) is obtained. Compared with GO, it was observed to present good conductivity due to the removal of chemical functional groups [90,91].

A comparative study was made between graphene and rGO to develop an SPR aptasensor for kanamycin detection. The aptasensor fabrication consisted of the functionalization of graphene or rGO on the gold layer followed by the binding of the aptamer to the sensor surface. For better covalent interaction, 1-pyrene butyric acid (BPA) was added to the graphene layer. The pyrene and carboxylic groups from PBA help the immobilization of the aptamer. The results show that chemical vapour deposition (CVD) of graphene provides more homogeneous sheets and immobilizes the aptamer better so that the antibiotic can be detected in the linear range of 1–100 µM [40].

Chiu et al. proposed an immunosensor using carboxyl-functionalized graphene oxide (COOH-GO immobilized onto a SAM of cysteamine on gold [92]. The activated COOH-GO reacts covalently with an amine available group from BSA or a protein ligand. Moreover, ethanolamine was added for blocking the remaining active sites from the surface of the biosensor. The refractive index of the modified chip varied with the wavelength (34.1 m°, 34.4 m° and 35.2 m° for 633 nm, 670 nm, and 690 nm). Antigen–antibody interaction was tested for GO and COOH-GO coatings, and it was observed that the shift angle was 9.13 m° for the Ab–BSA interaction with BSA/GO while for BSA/COOH-GO the obtained shift angle increased at 35.5 m°, revealing a good interaction with the biomolecules and a high surface area.

Wu et al. developed a biosensor for Pig IgG detection. They compared the performances of GO and COOH-GO materials [93]. For signal enhancement, AuNSt-antigen bioconjugates were used to interact with the Ab attached to GO or COOH-GO. It was observed that COOH-GO had more active sites and the Ab could be bound more efficiently compared with GO. The LOD of the proposed biosensor (AuNSt-antigen/COOH-GO) was 0.0375 μg mL^−1^.

**Figure 8:**
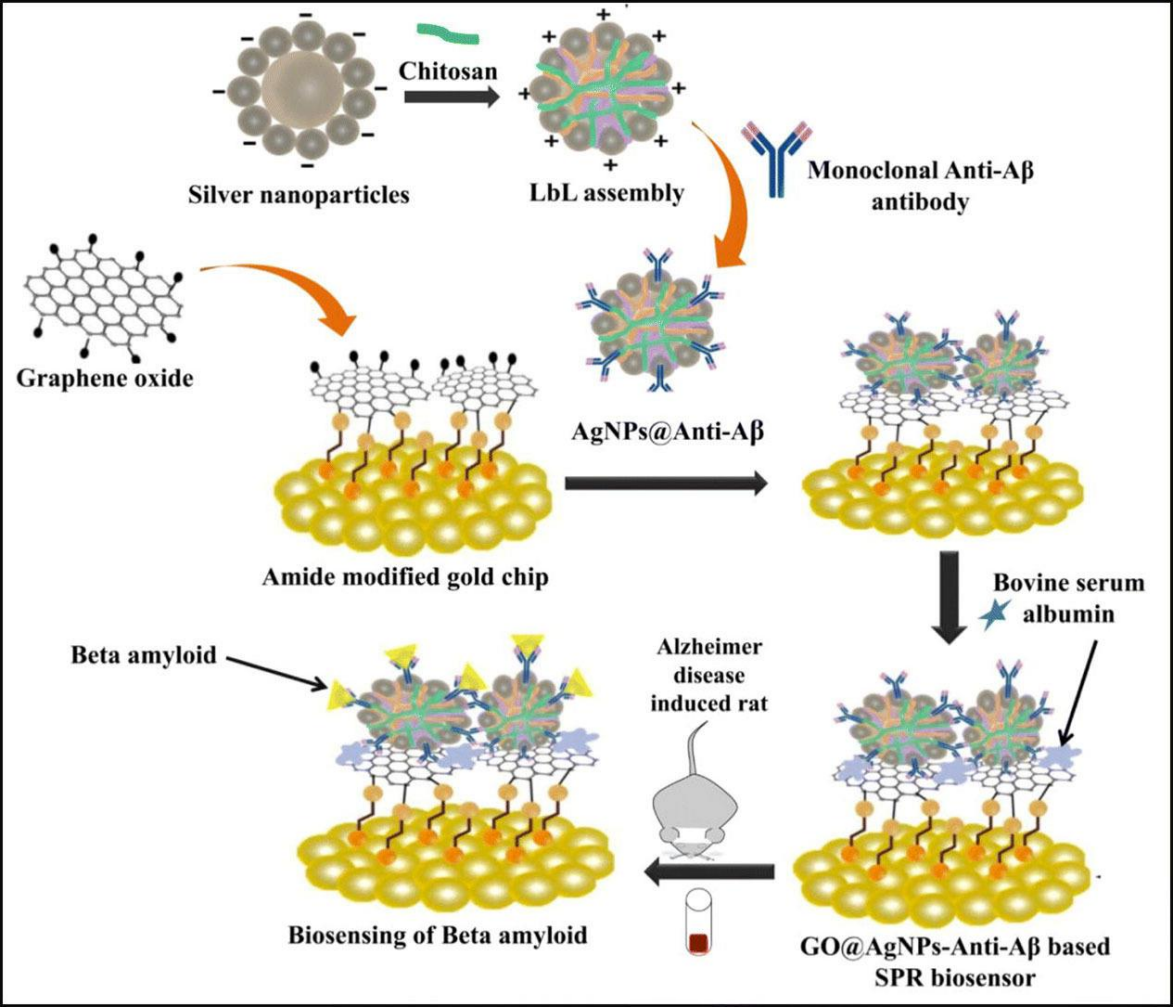
Fabrication of the Ab-β-amyloid/AgNPs-Cs-polystyrene sulfonate (PSS)/GO biosensor for detection of the β-amyloid biomarker. The gold chip was first functionalized with an amine group followed by immobilization of GO sheets via EDC/NHS reaction. LbL assembly was formed by three main components: AgNPs, chitosan (Cs, positively charged polymer) and polystyrene sulfonate (PSS, negatively charged polymer). Reproduced from [94] with permission of Elsevier.

Another biosensor for the β-amyloid biomarker was proposed by Nangare and Patil [94]: β-amyloid is a biomarker for the early diagnosis of Alzheimer’s disease. β-amyloid is a misfolded and insoluble peptide with a molecular weight of 4 kDa that develops cross-β-sheet super-secondary units [95]. It is assumed that this structure is resistant to proteolysis; layer-by-layer (LbL) assembly was used to obtain a controlled immobilization of the antibody. The main idea was to use cationic or anionic polymers to provide stable surfaces and efficient antigen/antibody binding. The schematic representation of the modified chip can be seen in Figure 8.

Besides modifying materials with graphene, the addition of MoS_2_ enhances the SPR signal due to its high optical and electrical properties [96]. MoS_2_ material has a hexagonal structure, formed of a hexagonal plane of S and one atom of Mo. Its structure facilitates the propagation of the surface plasmon, due to its large absorption coefficient at 500 nm and high refractive index, thus increasing the sensitivity. A modified carboxyl-MoS_2_ nanocomposite was used as a signal-amplifying sensing film for the detection of the lung cancer biomarker CYFRA 21-1. The carboxyl-MoS_2_ layer displayed good biocompatibility and affinity to the target analyte, showing a 355.69 m° increase in the SPR signal [97]. Nie et al. [98] proposed a biosensor composed of decorated MoS_2_-AuNPs for the detection of microRNA. It was observed that MoS_2_-AuNPs nanocomposite increased the SPR signal compared to the AuNPs alone. This behaviour could be explained by the large surface area provided by MoS_2_ for the attachment of AuNPs responsible for LSPR. DNA-linked AuNPs-MoS_2_ nanocomposite could detect microRNA with a limit of detection of 0.05 fM.

**Figure 9:**
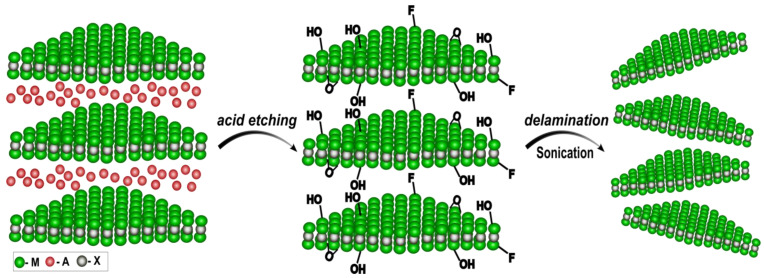
Preparation of MXenes by etching the A metal from the MAX phase, followed by exfoliation of the layers through sonication.

MXenes are compounds with similar structures to graphite (formed from several layers) that can be synthesized from MAX precursors: M = early transition metal, A = elements from groups 13 and 14, X = C, N. MXenes can be obtained after MAX etching (usually is used HF) (Figure 9). After the etching procedure, the A layer is removed and replaced with terminal functional groups such as -OH, -O, -F. Then MXenes are exfoliated to obtain 2D flakes [87,99].

**Figure 10:**
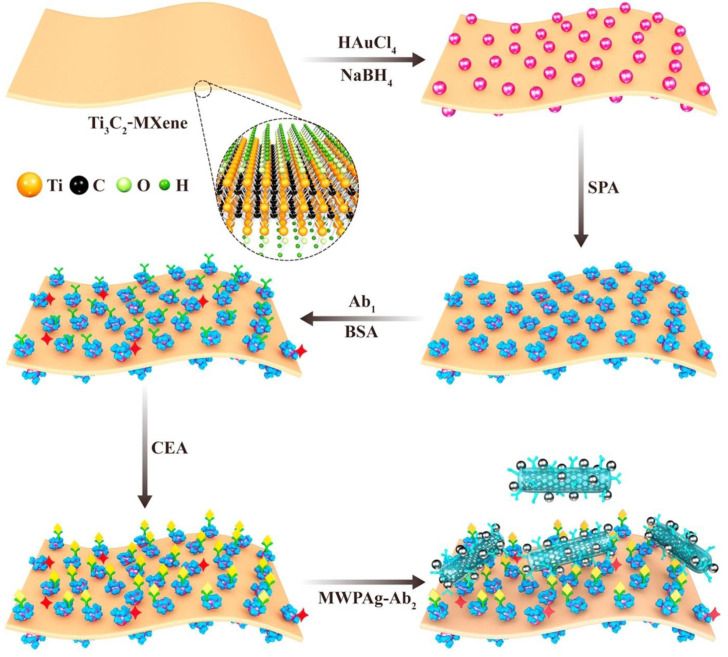
Schematic representation of the modified biosensor for CEA detection. A mixture of Ti_3_C_2_-MXene/AuNPs/SPA-Ab_1_On was prepared on the gold slide. CEA was injected in the following step. MWCNTs-Ab_2_ were added to complete the sandwich assay. Reproduced from [101] with permission from Elsevier.

A conventional SPR biosensor for detecting CEA (carcinoembryonic antigen) using MXenes layers was recently proposed [100]. The main structure of the biosensor included four substrates: first, amino-functionalized Ti_3_C_2_-MXene nanosheets were attached to the gold film. Hollow AuNPs (HAuNPs) were bound to these layers for further interaction with staphylococcal protein A (SPA). The last step was the conjugation of the specific Ab (which contained carboxyl groups) with the modified MXenes with amino groups. Different concentrations of CEA were analysed, and a linear range from 10^−9^ to 10^−15^ M was obtained. Another strategy for enhancing the SPR signal using MXenes, AuNPs and SPA was to add MWCNTs-polydopamine (PDA)-AuNPs modified with Ab_2_ in a sandwich assay for sensing CEA (Figure 10) [101]. At this time, multi-walled carbon nanotubes (MWCNTs) with a large surface area helped to attach the Ab_2_ in a more ordered position. In this case, the LOD for CEA was 0.07 fM.

Chen et al. developed an aptasensor to detect SARS-CoV-2 coronavirus [102]. They added Nb_2_C-MXene quantum dot (QD) homogeneous layers on the Au chip, forming a SAM via the thiol group from MXenes and Au. Subsequently, a specific aptamer was incubated on the modified chip for the detection of the N-gene of SARS-CoV-2. The main challenge was to test the specificity of the aptasensor in the presence of the interferences, which coexist with the N-gene. The biosensor showed high selectivity for the analyte in the presence of all chosen interferences, and the limit of detection was 4.9 pg/mL. The most relevant works reporting novel 2D nanomaterials for the development of SPR biosensors are summarized in Table 1.

While 2D nanomaterials are extensively used for signal amplification, it should be noted that in most SPR assays the protein/protein interactions are driven in a passive manner: the overall sensing time and sensitivity are dependent on the Brownian diffusion process, which greatly hinders their efficiency, especially at low concentrations. To overcome this limitation, an all-optical active method termed optothermophoretic flipping (OTF) was developed, and its efficiency was tested via antibody–antigen binding on an SPRi platform [103]. The OTF method is based on the thermophoretic force exerted on biomolecules or nanoparticles induced by temperature gradient. This study was based on an in situ thermophoresis-driven molecular interaction enhancement strategy, i.e., driving and enriching the solutes towards the vicinity of the sensing surface via different kinds of active external forces. The sensitivity enhancement factor of 23.6 was the highest enhancement factor achieved in existing SPRi assays.

## 6. Conclusions

Herein, we have summarized the most recent and relevant works on optical SPR detection and trending strategies for surface modifications. Among various strategies for anchoring biomolecules on the SPR chip, EDC/NHS coupling remains the most used immobilization method. Still, to achieve a high sensing performance, oriented immobilization of bioreceptors should be attained. For this purpose, biotin–streptavidin interaction, protein A/G–antibody interaction, His_6_-tag protein/NTA/Ni^2+^ (Cu^2+^) reaction and click reaction are extensively used. Current strategies combine these types of immobilizations in various formats to increase the surface coverage of the anchored biomolecules. The implementation of conventional SPR biosensors in portable and miniaturized devices is challenged by the fact that an isothermal regime must be maintained since the temperature fluctuations greatly influence not only the SPR response but also the strength of affinity and the kinetics of the binding. On the contrary, the LSPR biosensors are not temperature sensitive because LSPR sensing is based on absorbance measurements and is carried out using common laboratory equipment; moreover, LSPR can significantly decrease the mixing time, since the sample distributes faster to the surfaces of the nanoparticles than to the metallic film.

One of the major drawbacks of the SPR systems remains the analysis of the compounds from a complex matrix. As the electrostatic interaction represents the main interaction occurring on the surface of an SPR sensor, the extent and sign of the electrostatic interaction between charged biomolecules can be tuned within a relatively wide range of pH values. Increased ionic strength screens charged groups and has a pH-independent repulsion effect on hydrophilic and charged surfaces due to the formation of ion formation that neutralizes the charged domains [104]. At low ionic strengths, the pH becomes increasingly significant as the electrostatic interactions are driven by the ligand’s overall charge, which is positive at pH values below the ligand’s isoelectric point (pI) and negative above pI. A pH near the pI of the interacting species minimizes the extent of electrostatic forces, increasing the influence of hydrophobic interactions. Therefore, the precipitation of proteins may often occur at the pI because of the weak electrostatic repulsion between biomolecules. Reducing the non-specific interaction and fouling behaviour, materials such as PEG, zwitterionic compounds and others were used to form a protective layer against interfering proteins but also served to interact further with the bioreceptor. Platforms employing nanocomposites and 2D materials provide unprecedented advancement in signal amplification and operational stability, reducing mixing times and providing low detection limits in SPR assays.

## Figures and Tables

**Table 1:** Design and performance of 2D material multilayer SPR biosensors.

Coating Biomaterial	Target	Sensitivity (Deg or RIU)	LOD	Dynamic Range	Ref.
Aptamer/CVD graphene	Kanamycin	2.1·10^−3^ RIU	0.28 µM	1–100 µM	[40]
Aptamer/rGO		1.9·10^−3^ RIU	1.79 µM	5.88–100 µM	
BSA/COOH-GO	Ab-BSA	35.5 m°	0.01 pg/ml	0.01–100 pg/mL	[92]
BSA/GO		9.13 m°	-	-	
AuNSt-antigen/COOH-GO	pig IgG	-	0.0375 μg/mL	0.0375–40 μg/mL	[93]
Ab-β-amyloid/AgNPs-Cs-PSS/GO	β-amyloid protein	280 RIU	1.21 fg/mL	2 fg/mL–400 ng/mL	[94]
Ab-CYFRA 21-1/COOH-MoS_2_	CYFRA 21-1	355.69 m°	0.05 pg/mL	0.05 pg/mL–100 ng/mL	[97]
DNA/MoS_2_-AuNPs	microRNA-141	193 m°	0.05 fM	0–50 pM	[98]
Ab-conjugated SPA/HAuNPs/N–Ti_3_C_2_-MXene	Carcinoembryonic antigen	-	0.15 fM	0.001−1000 pM	[100]
MWCNTs-(PDA)-AuNPs-modified with Ab_2_/Ab_1_-conjugated SPA/AuNPs/Ti_3_C_2_-MXene	Carcinoembryonic antigen	369 m°	0.07 fM	2 × 10^−16^–2 × 10^−8^ M	[101]
Aptamer/Nb_2_C-MXene-QDs	N-gene of SARS-CoV-2	270 RIU	4.9 pg/mL	0.05–100 ng/mL	[102]

## Data Availability

Not applicable.
